# How do Workers with Common Mental Disorders Experience a Multidisciplinary Return-to-Work Intervention? A Qualitative Study

**DOI:** 10.1007/s10926-014-9498-5

**Published:** 2014-02-15

**Authors:** Malene Friis Andersen, Karina Nielsen, Svend Brinkmann

**Affiliations:** 1 National Research Centre for the Working Environment, Lersø Parkallé 105, 2100 Copenhagen ∅, Denmark; 2 Work and Organisational Psychology, Norwich Business, University of East Anglia, Norwich Research Park, Norwich, NR4 7TJ UK; 3 Department of Communication and Psychology, University of Aalborg, Kroghstræde 3, 9220 Aalborg, Denmark

**Keywords:** Sick leave, Mental disorders, Intervention, Rehabilitation, Return to work, Qualitative research

## Abstract

*Purpose* Long-term sick leave due to common mental disorders (CMD) is an increasing problem in many countries. Recent reviews indicate that return to work (RTW) interventions have limited effect on reducing sickness absence among this group of sick-listed. The aims of this study were to investigate how sick-listed persons with CMD experienced participating in an RTW intervention and how workability assessments and RTW activities influenced their RTW-process, and to examine the working mechanisms of the intervention. The gained knowledge can help improve future RTW intervention design and implementation. *Methods* In-depth interviews were conducted with 17 participants on sick leave due to CMD who participated in an RTW intervention. Interviews were conducted at three time points with each participant. Principles of interpretative phenomenological analyses guided the analysis. *Results* The workability assessment consultations and RTW activities such as psychoeducative group sessions and individual sessions with psychologist could result in both motivation and frustration depending on the extent to which the RTW professionals practiced what we have termed an individual approach to the sick-listed person. *Conclusions* The individual approach seems necessary for the realization of the positive potential in the RTW intervention. However, the fact that RTW professionals are both the facilitators and the controllers of the sick-listed persons’ RTW process is an inherent paradox in the intervention, which can impede the necessary establishment of a high-quality relationship between the sick-listed persons and RTW professionals.

## Introduction

Long-term sick leave due to common mental disorders (CMD), such as depression, anxiety and stress-related disorders is an increasing problem in many countries [1–6]. Long-term sick leave is a major risk factor for early withdrawal from the labour market, and only 50 % of those off work for more than 6 months due to poor mental health return to work [7]. CMD make up an increasing percentage of claims for disability benefits [6, 8]. CMD-related sick leave and withdrawal from the labour market is not only costly for society and workplaces due to compensation costs and lost productivity [9], being off work also frequently has negative consequences for the individual, because work is socially highly valued and beneficial to self-respect, identity, health and general well-being [10]. To reduce both the human, societal, and economic consequences of long-term sick leave due to CMD, a better understanding of the factors that either facilitate or complicate Return To Work (RTW) for employees with CMD is warranted.

In recent years there has been an increasing interest in research on the effectiveness of RTW interventions for employees on sick leave due to CMD. Three reviews on the effects of RTW interventions for persons on sick leave due to CMD have been published in the last few years [11–13]. The review by Arends et al. found positive results for partial RTW for RTW intervention based on problem solving therapy, but not for interventions based on cognitive behavioral therapy. Neither of the two types of interventions succeeded in decreasing the time to full RTW compared to usual care [11]. Pomaki et al. [13] concluded in their review that workplace-based interventions can improve work disability outcomes defined as (1) work absence duration, (2) work functioning, (3) quality of life, and 4) economic outcomes for persons with CMD. The effects were highest for the last three outcomes and only one study showed moderate evidence for reduced work absence duration. The third review included studies on the effect of RTW interventions for persons on sick leave irrespective of their specific medical diagnoses [12]. Hoefsmit et al. [12] concluded that early interventions (less than 6 weeks’ sick leave) and multidisciplinary interventions support RTW for all medical conditions, but interventions addressing physical health problems were more effective than those addressing mental health problems [12]. The general conclusion based on the three reviews seems to be that RTW interventions for workers on sick leave due to CMD have no or only a limited effect on time to full RTW. The reviews mostly explain the limited effect by referring to methodological issues such as small numbers of participants in the studies and high-quality of usual care [11–13].

The majority of studies on RTW interventions are exclusively evaluated by quantitative methods focusing on specific outcomes such as time to RTW, severity of symptoms, work functioning, etc. While quantitative studies are optimal for investigating the effects of RTW interventions, they are not suitable for capturing the complex processes characterizing the experience of returning to work [14–16]. The increase in sick leave due to CMD and the limited effect of RTW intervention point to important questions that are so far unanswered. In this article we want to investigate:

- How do persons on sick leave due to CMD experience workability assessments?
- How do persons on sick leave due to CMD experience the RTW activities offered?
- What are the working mechanisms of the RTW program and which underlying dynamics of the RTW program influence the sick-listed persons’ RTW-process?

Qualitative research can help us shed light on these questions and thereby contribute to interpretations of quantitative findings. A meta-synthesis of qualitative research on RTW for employees on sick leave due to CMD identified and included eight studies on sick-listed persons’ experience of the RTW-process [14]. The studies included focused more broadly on general obstacles to and opportunities of returning to work. Only a few studies focused somewhat on the sick-listed persons’ experiences with a concrete RTW intervention [14]. In relation to the last topic the studies pointed to the importance of receiving flexible and continual support from an occupational therapist that also coordinated the RTW-process with relevant stakeholders [17–19]. We have limited knowledge of how persons on sick leave due to CMD experience participating in an RTW intervention and how specific elements and activities in an RTW intervention influence their RTW-process. This knowledge, however, is important to further tailor RTW interventions to the needs of persons sick-listed with CMD. The aim of this qualitative study was to explore how sick-listed persons with CMD experienced participating in the largest randomized RTW intervention in the world [20, 21]. The RTW intervention is described in detail below.

This article is based on a longitudinal qualitative interview study conducted with 17 workers on sick leave due to CMD, who participated in an RTW intervention carried out in Denmark from April 2010 to April 2012 (Henceforth referred to as the RTW program). The RTW program was implemented in 22 municipalities in Denmark and approximately 13.000 workers on sick leave regardless of medical diagnosis participated in the intervention [22]. A Danish report on the preliminary results of the project concluded that on the whole the RTW intervention did not succeed in reducing sick leave, however, there were great variations across municipalities [22].

## Materials and Methods

### Design and Theory

Theoretically this study is inspired by Interpretative Phenomenological Analysis (IPA) (see the section Analysis) developed by Jonathan Smith [23, 24]. IPA is founded on phenomenology (being an approach to the study of human experience), hermeneutics (the theory of interpretation) and symbolic-interactionism (focusing on the meanings people attach to situations, which can only be accessed through interpretation) [25]. IPA stresses the importance of interpretation and is founded on the idea that the inner world of a person is reachable through qualitative inquiry: “it [IPA] assumes an epistemological stance whereby, through careful and explicit interpretative methodology, it becomes possible to access an individual’s cognitive inner world.” [25]. Our study draws on assumptions from post positivism (we acknowledge that the sick listed workers are actually experiencing “real” symptoms) as well as from constructionism (we assume that *how* they understand and interpret their symptoms is influenced by cultural meanings and discourses) [26].

Three semi-structured interviews were conducted with each of the 17 participants recruited for this study. Our study addresses the call of Andersen et al. [14] for longitudinal qualitative research investigating the RTW-process from the perspective of the person on sick leave. Conducting three interviews with each participant over an extended period of time (6–7 months) enabled us to hypothesize on relationships between different subjects and provided information on whether the participants’ experience and perception of the RTW program, their health problem and work situation changed over time. Besides, the prospective research design enabled us to explore the participants’ immediate experiences, feelings and decisions while the outcome of their situation was still uncertain.

### Participants

Participants were recruited when randomized to the Danish RTW program. The inclusion criteria for this interview study were (1) the participant stated that she or he was on sick leave due to stress or depression, (2) the participant was randomized to the RTW program after being on sick leave for approximately 8 weeks, (3) the participant spoke and understood Danish. Two recruitment strategies were used: (1) Recruitment by letter: Twice a week invitation letters were distributed to individuals matching the inclusion criteria from six municipalities; and (2) Recruitment face-to-face: Caseworkers in one of the six municipalities identified workers on sick leave matching the inclusion criteria and invited them to receive more information on the study from the first author in an office next door. Even though we aspired to get a homogenous sample, the lack of an official definition of stress as a disorder [27, 28] had as a consequence that the sick-listed workers who reported stress as the cause of their sick leave often had a number of symptoms identical with those related to anxiety disorders and adjustment disorder. Therefore we found it more appropriate to define the participants more broadly as being on sick leave due to common mental disorders and not narrowly on leave due to stress or depression.

### Setting

The RTW program consisted of an early, multidisciplinary, and coordinated effort within the existing legal framework and under the management of the municipal sickness benefit offices. Each municipality established at least one multidisciplinary unit consisting of RTW coordinators (municipal sickness benefit officers), RTW teams (psychologists and physiotherapists/ergo therapists), and clinical units (psychiatrists and physicians from occupational, social or internal medicine) (Henceforth referred to as RTW professionals). The RTW program was aimed at persons on sick leave categorized by the sickness insurance offices as having complex health related problems irrespective of medical diagnoses.

The person on sick leave participated in a meeting with the RTW coordinator before the end of the eighth week of sick leave. At the first meeting, the RTW coordinator used a standardized assessment tool that identified resources and barriers for RTW related to physical and mental health, work, and occupational situation. On the basis of the gained information, the RTW coordinator decided if involvement of the RTW team and clinical unit was needed. In more complex cases (approximately 50 % of the cases) the RTW coordinator had to involve the RTW team for further workability assessment and in the most complex cases the clinical unit was involved (approximately 25 % of the cases). The multidisciplinary unit held weekly meetings where they coordinated and discussed cases and decided on a tailored RTW plan suggesting relevant RTW activities for the person on sick leave. According to Danish law, members of the multidisciplinary team are not allowed to offer traditional treatment such as psychotherapy. Instead the offered RTW activities typically consisted of psycho-educative group sessions, a few individual sessions with the psychologist, physical exercise, and meetings with the workplace (for more details on the intervention see [20, 22] ).

**Fig. 1:**
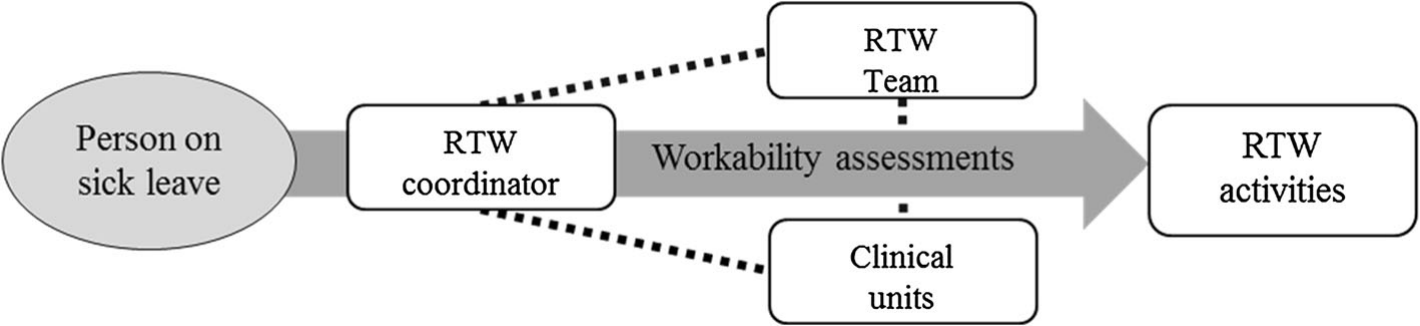
Pathways in the RTW program

Figure 1 shows the possible pathways for the person on sick leave when participating in the RTW program.

The sick-listed persons’ participation in the intervention was not voluntary. According to the law, workers on sick leave have to participate in workability assessments and RTW activities offered by the social insurance office if the RTW coordinator estimates that it would enhance the chances of returning to work. If they refuse to do so, their sickness benefits could be withdrawn. It is important to take this legal context into account to understand the conditions under which the persons on sick leave experienced their RTW-process [14].

### Data Collection

The first interview was conducted just after the randomization to the RTW program, the second interview was conducted approximately 3 months after the first interview and the third interview 6–7 months after the first interview. This research design made it possible for us to follow the participants over an extended period of time. The first interview was conducted before the beginning of the intervention and enabled us to explore how the participants experienced being on sick leave due to CMD. This knowledge is important in order to understand the background of the participants as it can influence how they experience and interpret the RTW program.

The content of the interview guides was the same for all three interview sessions for most themes, however, in relation to the RTW intervention the first interview addressed the expectations, hopes, needs and fears in relation to the intervention offered by the municipality and the RTW-process, whereas the second and third interviews addressed the actual experience of the RTW program. Furthermore, at the first interview the interview guide included a number of questions concerning background information of the sick listed worker and the period up to the onset of the sick leave.

The interviews were all conducted by the first author (psychologist). They lasted between 1 and 2.5 h and were all audiotaped and transcribed verbatim. Interviewees decided where the interview took place. Most of the interviews were conducted in the homes of the sick listed persons. A few were conducted at the first author’s workplace, one interview at the workplace of one interviewee and another interview was conducted in a meeting room in a public library. Three interviews were conducted by phone due to practical problems arranging the interviews face-to-face. The interview guide was tested in a pilot phase and the final version of the guide comprised overall themes such as: Illness representation, work situation, perceptions of barriers and resources for RTW, experience of the RTW program, appraisal of the influence of the RTW program on the health problem and RTW-process. The interview guides were explorative apart from the investigation into the theme “illness representation” which was partly theory-driven [29–37]. In Denmark an approval from Ethical Committee was not required as this study did not include biomedical research, but the study was registered with the Danish Data Protection Agency [38] and we followed the ethical guidelines provided by the British Psychological Society. It was stressed in the beginning of every interview that participation was voluntary and that the participants were free at any time to withdraw from the study. Full anonymity was guaranteed and all information was kept confidential. In the invitation letter and at all three interviews the sick listed workers were informed that refusal to participate in or withdraw from the interview study would have no influence on their claim to sickness benefit nor on the intervention offered.

### Analysis

The study is phenomenological in a broad sense, i.e., with an interest in the lived experiences of the participants, but it deviates from the strict phenomenological methodology developed by Amedeo Giorgi [39], for example, and is instead in line with principles from Interpretative Phenomenological Analysis (IPA) developed by Smith and colleagues [23, 24]. IPA is inductive and concerned with exploring a person’s lived experiences and how he or she makes sense of important transitions and decisions in life [23, 24]. The method is suited to studies that aim to relate findings to bio-psycho-social theories [25] and it has shown its relevance in exploring the psychological and dynamic processes characterizing individuals’ experiences of sickness and reduced functional level [23, 40–42]. Due to the large amount of data, a slightly adapted version of IPA was applied: The 51 interviews were all read and re-read by the first author and units of meaning were identified as central aspects of the participants’ experiences and narratives. On the basis of this identification, themes and categories were developed and all 51 interviews were coded in the qualitative data analysis software NVivo 9 (QSR International Pty Ltd., Version 9, 2010) in accordance with these themes and categories. Apart from the codes related to illness representation [34, 43], which is not presented in this article, all codes and categories were data driven and emerged from the material. The codes were adjusted if needed and the interviews were re-coded if any adjustments were made. Mind maps of the themes, categories and their interrelatedness were developed by the first author and these were discussed and changed until consensus was obtained by all three authors. In this article we focus on emergent key themes for the whole group. In line with the IPA recommendations for working with large samples we have focused on summarizing and condensing the main themes [24]. Therefore the analysis and presentation of each case will inevitably be less detailed—but still detailed enough to ensure that the identified group level themes can be illustrated with particular examples from the cases [24].

IPA stresses the importance of the researchers knowing their own fore-conception (prior experiences, assumptions, preconceptions) of the subject under scrutiny [24]. To meet this demand a high degree of reflexivity in the research process is required. Accordingly, the first author received supervision from an external clinical psychologist during both data collection and data analysis in order to secure a constantly nuanced level of reflection as well as awareness of her own role as a researcher and her own personal bias. Furthermore, during the research project the three authors discussed and reflected on selected material in relation to their own fore-conceptions.

## Results

### Participants

Invitation letters were sent to 93 potential participants and approximately 20 % of those invited accepted to participate. For ethical reasons we did not explore the reasons for refusal to participate due to the vulnerability of the sample. A total of 18 participants were recruited. One participant withdrew after the first interview. The participant who withdrew first agreed to participate in the second interview but when we tried to arrange a time for the conduction of the second interview she did not reply to a number of emails and phone calls. We do not know the reasons for this.

Three interviews were conducted with the remaining 17 recruited participants (13 women and 4 men). This article is therefore based on 51 interviews. The average age of the participants was 44 years (range 23–61 years). Nine were on sick leave due to self-reported stress and 8 due to self-reported depression. The participants were employed in various occupations such as manual work, people-related jobs and knowledge work. At the last interview session, 11 participants had returned to work full time or part time or were no longer on sick leave (Some of the participants were laid off during their sick leave and therefore had no job to return to. When the authorities declared them able to return to work or they did so themselves they would typically receive sickness benefit or social welfare and be obliged to apply for a new job). Six participants were still on sick leave at the last interview session (See Table 1 for detailed information on the participants).

**Table 1:** Participant description

Participant	Sex & Age	Reason for sick leave	Work status at the three interviews
1	M, 39	S	1:FS, 2:PR, 3:FR
2	F, 40	S	1:FS, 2:FR, 3:FS
3	F, 32	D	1:FS, 2:FS, 3:FS
4	F, 47	S	1:FS, 2:PR, 3:PR
5	F, 59	D	1:FS, 2:FS, 3:FS
6	F, 55	S	1:FS, 2:FS, 3:FS
7	F, 49	S	1:FS, 2:FS, 3:O-E
8	M, 38	D	1:FS, 2:O-E, 3:O-E
9	M. 31	D	1:FS, 2:FS, 3:FS
10	F, 55	S	1:FS, 2:PR, 3:PR
11	F, 49	D	1:FS, 2:FS, 3:FS
12	F, 55	S	1:FS, 2:PR, 3:FR
13	F, 48	D	1:FS, 2:O-E, 3:O-E
14	F, 23	D	1:FS, 2:O-E, 3:FR
15	F, 43	S	1:FS, 2:PR, 3:FR
16	F, 26	S	1:FS, 2:O-E, 3:FR
17	M, 61	D	1:FS, 2:FR, 3:FR

*M* male,* F* female,* S* stress,* D* depression,* FS* full time sick leave,* PR* partial RTW,* FR* full RTW,* O-E* off the sick list but unemployed

### Participants’ Perceptions of the RTW Program

We will present our analysis of the interviews under three headlines: Persons with CMD’s experience of workability assessment, Persons with CMD’s experience of RTW activities, and Working mechanisms of the RTW program. The analysis of the interviews revealed three categories in relation to each of the main themes. Under the headline Persons with CMD's experience of workability assessment the following three categories will be presented: 1. Participants’ uncertainty about the aim of the assessment consultations, 2. The difficulty of verbalizing one’s mental condition, 3. Fear of intensification of symptoms. Under the second headline Persons with CMD’s experience of RTW activities the following three categories will be presented: 1. Few individual sessions with RTW psychologist, 2. Psycho-educative group sessions, 3. Inadequate RTW activities. Under the last headline Working mechanisms the following three categories will be presented: 1. Individual approach, 2. RTW professionals as legitimate experts, 3. Multidisciplinarity. In the section Discussion below we will present a model showing possible interrelatedness between the themes in order to qualify our understanding of how and why the RTW program manages or fails to improve the chances of RTW for people on sick leave due to self-reported stress or depression.

Before going into detail with these themes, we will situate the participants of our study. It is important to understand their perception of and experiences with being on sick leave and their understanding of their medical condition as well as their feelings as these aspects seem to influence their perception of the RTW program and its success in meeting their needs.

#### Being on Sick Leave Due to CMD

The participants had different social, economic and work related backgrounds, and the major causes of the development of the mental health problem and sickness absence also seemed to differ. Still, they shared a number of common features and conditions related to the experience of being on sick leave due to CMD.

Almost all participants reported symptoms such as concentration problems, memory problems, feelings of inadequacy, self-reproach, low self-esteem, low energy, negative thinking. They experienced considerable and unpredictable fluctuations of symptoms, which made it difficult for them to estimate the state of their mental condition, and, consequently, when and how to return to work. They all found it difficult that their health problem was invisible and diffuse, and they lacked certain knowledge about when they had recovered. Without this knowledge it was difficult for them to navigate and make decisions about RTW:

I have not broken a leg, that’s true, I have not been operated on, that’s true too. But actually, I would have preferred that. Because that heals. But this is not easy, because you don’t know if it heals, and if it heals how much is lost and broken, anyway? It is difficult for people to understand, measure and see, because it is not concrete. Then it becomes diffuse, and then you become insecure. (Interview 1 with participant 11, woman, 49, on sick leave).

The majority of the participants was ashamed of their CMD and saw it as a personal defeat to have to report sick and no longer be able to cope with normal, everyday activities. Often they found their CMD and their sickness absence both illegitimate and unacceptable:

You feel really, really bad about being on sick leave. At least I do. I felt enormously guilty about letting everybody else down. Although the people I had worked with hadn’t treated me nicely I think: ‘It will be hard for them, the children will suffer, and what will the parents think?’ You have a lot of thoughts because somehow it is a major defeat to have to say: Okay, now I simply have to report sick because I have had it. (Interview 1 with participant 14, woman, 23, on sick leave).

For many of the participants, it was their first experience with a mental health problem, and for many a number of negative occurrences preceded the development of their CMD. Having developed a mental health problem, and being on subsequent long-term sick leave, led to a feeling of existential disturbance of identity: One no longer knows for sure who one is.

#### Persons with CMD’s Experiences of Workability Assessments

In this section we will present our findings in relation to our first research question on how the workability assessments were experienced. The participants reported different experiences with the assessment consultations with the RTW team. The participants who had positive experiences with the assessment felt that it helped create structure and direction in their somewhat chaotic and uncertain situation. Furthermore, they felt it enhanced their knowledge of their health situation and of how and when to return to work. A participant sick-listed with depression and with former long-term sickness absences described the importance of the assessment for him:

It was her (a RTW psychiatrist) who found out I had Asperger’s Syndrome. It has helped a lot, because now I know what is wrong, and now I know why I am as I am. It makes it easier for me to change some things.”(Interview 2 with participant 9, man, 31, on sick leave). He later elaborated on the importance of the assessment: “I can see that I need special conditions and special things in order to be a reliable worker at a workplace. And now I know what things.

This participant was unaware of the fact that he had an undiagnosed Asperger’s syndrome. For this participant, the assessment consultations with the RTW psychiatrist provided him with knowledge of how to compensate for and handle work related consequences and barriers associated to his Asperger’s syndrome and thereby prevent repeated depression and sickness absence. On the basis of this new knowledge and insight he had discussed concrete demands of a future workplace with the RTW professionals.

But participation in the assessment consultations could also create frustration and uncertainty in some of the participants. The negative experiences should be understood in the light of the characteristics of CMD and were mainly related to: (1) uncertainty about the aim of the consultations, (2) trouble verbalizing one’s problems and condition, and (3) fear of intensification of symptoms.

##### Participants’ Uncertainty About the Aim of the Assessment Consultations

Several participants failed to see the purpose of the RTW coordinator referring them to consultations with the RTW team or clinical unit. A person sick-listed with depression described the difficulty of decoding the purpose of the assessment consultations:

It is all terribly confusing for me, and it took me a long time to figure out what was actually happening [in the assessment consultations]. I didn’t understand it because I have concentration difficulties. Especially if I am out, then it feels like my senses are so busy with everything around me from the coffee pot to what others are saying, and I become, like completely….If you imagine a lot of different music in your head at the same time. (Interview 2 with participant 11, woman, 49, on sick leave)

The difficulty of deciphering and understanding the aim of the assessment consultations may be explained by the fact that a number of the core symptoms reported by the participants such as reduction of executive functions seem to reduce the ability to decode the purpose and tasks of the different RTW professionals. The confusion about the aim may, furthermore, be increased by the fact that the participants experienced that the RTW professionals had not always been sufficiently explicit about the aims of the consultations.

A third factor that seemed to create uncertainty about the aim of the assessment consultations was that some of the participants were already in contact with other health practitioners (typically psychologists and physicians). These practitioners were often of the utmost importance to the sick-listed persons, their conception of their condition and of the compatibility between the job and their recovery. If the participants felt well taken care of by other practitioners they sometimes failed to see the relevance of being referred to and assessed by the RTW professionals:

- Interviewer: *Do you know why you had a consultation with the RTW psychologist?*
- Participant: *It was something that the RTW coordinator decided. I don’t know what was the big idea…I think “Why does* [the municipality] *waste money on an hour with a psychologist when I have already seen another psychologist ten times, who is much, much* *better* *than the RTW psychologist?”* (Interview 2 with participant 8, man, 38, off the sick list but unemployed).

#### The Difficulty of Verbalizing One’s Mental Condition

A number of participants found it difficult to describe their situation and their mental condition during the assessment consultations with the RTW professionals:

When I was referred to the municipality [the RTW professionals] I thought: How do I explain how I feel? I mean, it is really difficult, because you can’t tell by looking at a person, can you?” (Interview 1 with participant 13, woman, 48, on sick leave). And later she explains: “there are some things you can’t do, and I can’t show them I can’t do it. If you have one arm, you can show that you can’t do the dishes. But I can’t - it is so difficult to sit there and explain that inside you….that there are things you can’t do.

It frustrated some of the participants that they were unable to produce “objective” proof of their health problem or reduced workability, and they found it difficult to state precisely what exactly they could or could not do during the one hour set aside for the consultation. Besides, some participants questioned the ability of the RTW professionals to judge competently on the basis of one single consultation how ready they were for work, which RTW activities they needed, and if they were entitled to sickness benefit:

So I feel, like, how are they [the RTW professionals] going to judge how well they think I am? In fact only myself and my doctor and my psychologist know that. I think it is difficult for people who don’t know me at all to say: Now I’ll just check your health and - Well, we think you are well enough. (Interview 1 with participant 14, woman, 23, on sick leave)

Some participants were convinced that relatively intimate knowledge of a person and his or her inner dynamics and outer world is needed to be able to give an opinion of the seriousness of the mental health problem, workability and need for RTW activities.

Some participants experienced that RTW professionals expected a comparatively concrete description and explanation of their situation and its cause plus an estimate of when they were ready for RTW:

During those consultations it’s very much like, “when are you able to return to work?” And I say,. “I don’t know”, “Well, but why are you depressed?” But I don’t know either. They expect a concrete description of why things are like they are and when you expect to return. I mean, if it was up to them they would like an exact date and time for my return. And that’s really, really difficult when it is a mental thing. (Interview 2 with participant 14, woman, 23, off the sick list but unemployed).

Many experienced that their symptoms fluctuated: One day they felt they shirked work—because they felt fit for working—and the next day they might break down crying at the thought of having to confront their present or a future workplace. The fluctuation of symptoms and the often complex causes of the CMD made it difficult for the participants to provide the precise and concrete answers that they felt the RTW professionals expected.

#### Fear of Intensification of Symptoms

For some of the participants a number of negative occurrences and experiences preceded the development of the CMD and sickness absence, and, as mentioned above, some were ashamed of being sick-listed with a mental health problem. The assessment consultations could be emotionally demanding as verbalization of the past and the CMD for a few participants seemed to actualize negative feelings and experiences:

I had just been telling the psychologist that I hated to have to tell it all again [to the RTW physiotherapist]. Because I also broke down when I talked to him. I told him there were some things I found difficult to talk about. Because it is a total failure. It is terrible to talk about oneself in that way…so it was…bloody hard. (Interview 2 with participant 13, woman, 48, off the sick list but unemployed).

The fear that the consultations would intensify symptoms might even discourage a few participants from describing their condition and situation. One participant related how she *“shut up like a clam*” in front of the RTW psychiatrist because she was afraid that she would not be able to escape the chaos that might arise during the retelling of her situation, her symptoms and experiences.

Altogether, according to the participants, the assessment consultations with the RTW professionals seem suited to perform workability and health assessment. But for them the nature of mental health problems, and the experience of being sick-listed because of these, call for special attention to how the assessment consultations are introduced and conducted.

#### Persons with CMD’s Experiences of RTW Activities

In this section we will present our findings in relation to our second research question on how the offered RTW activities were experienced. The focus will be on’few individual sessions with psychologist’ and ‘psycho-educative group sessions’, which were the most common RTW activities offered to the participants. Both activities were based on principles from cognitive behavioral approach, which is the most frequently used approach in psychological RTW interventions [44]. Finally we will present our findings in relation to the participants’ experience of ‘inadequate RTW activities’.

##### Few Individual Sessions with RTW Psychologist

The participants were generally satisfied with the consultation with the psychologist and they found the work-related focus of the consultation useful. Participants with comparatively minor health problems mentioned benefiting most from the consultations. According to the Danish sickness benefit legislation, the RTW psychologist was not allowed to offer actual treatment, and the participants, accordingly, frequently described the consultations as advice or guidance. If the situation of the participant was complex, mentally as well as socially, the result of the relatively few consultations was perceived to be of limited use to the participant. One participant, sick-listed with stress and with both work-related and complex social problems, described how the three consultations she had been offered were insufficient for the psychologist to be able to intervene:

The physiotherapist had a very clear focus on my body in relation to my job. But with the psychologist, we didn’t quite manage, because it is not just something that happens during two consultations. Not at all. It is easier to relate to your body, how it functions, how your joints are. You can feel that. But with your mind - it is different. Stress and such, I think it is very vague and diffuse…..There are many things you have to deal with and solve. (Interview 2 with participant 2, woman, 40, returned to work).

This participant was sick listed again at the third interview as her mental health problem and social situation had deteriorated. A few participants found it unsettling and confusing that no traditional treatment was offered. If the consultations had identified and clarified central problems (e.g. additional diagnosis or problematic personality traits) the participants felt abandoned without help or tools to cope with the problems disclosed to them during the consultations.

##### Psycho-Educational Group Sessions

The participants who were offered psycho-educational group sessions found the offer relevant and helpful. In particular they appreciated that they had gained knowledge of the interconnection of body and mind, and also that they had developed a new framework for understanding their symptoms and had been inspired to apply new coping strategies when returning to their former job or to a new one:

It was great to be told that it was part of the illness that the illness affects your memory and concentration, because it is the body’s way of shutting down the system….So in a way it has been good to be told that there is a natural explanation. In the beginning I thought I was going crazy. (Interview 2 with participant 15, woman, 43, partial RTW).

The participants felt put at ease about their physical and mental symptoms, which some feared were chronic or downright life-threatening. E.g. some feared that arrhythmia was a possible heart attack. In the psycho-educational group sessions, the RTW professionals may be characterized as co-interpreters of symptoms, a role which seems to be very useful for persons with CMD. As mentioned above, the health problem often appears diffuse and indefinite and the new knowledge gained may enable the participants to see work as compatible with their symptoms and to orient themselves towards RTW. The participants also emphasized the advantage of being with other sick-listed persons in the same situation:

My God, am I the only one or does anybody else feel the same? Then we sit there talking about it, well, and then, oh God, we are not alone. Then I am not the only one, and then you are not the only one. (Interview 2 with participant 5, woman, 59, on sick leave).

Being with others in the same situation seemed to normalize the condition of the participants, restored their self-confidence and reduced the feeling of being alone. Several participants stressed that it was decisive for the positive outcome of the group session that the other participants had identical or similar health problems. The interviewed participants who were not offered psycho-educational group sessions were most often not informed by the RTW professionals of the existence of this specific RTW activity and could therefore not ask for permission to participate in this—even though in the research interviews some expressed a need for intervention similar to the group sessions.

##### Inadequate RTW Activities

Not all participants had taken part in RTW activities after the assessment consultations. Sometimes the absence of activities agreed with the participants’ own sense of not needing or having the energy to participate in an RTW activity. Sometimes, however, activities were absent but were seen as needed, and one or more of the RTW activities contained in the RTW program might have met these needs:

I would have liked somebody to ask me “How do you feel about being back?” and who said, “You must change your way of thinking, and what would you do to change things?” Being challenged a little more and then receiving a few consultations. Maybe they [RTW actors] could coordinate with my workplace and say:” What can we do to help him not to get himself in the same hole again. (Interview 2 with participant 8, man, 38, off the sick list but unemployed).

Based on the interviews conducted with the participants we assume that there may be an association between a lack of offers of either individual consultations, psycho-educational group sessions or contact with the workplace and an increase in the risk of recurrent sickness absence or aggravation of mental health problems for a few participants. The participant quoted above, for example, was reported fit for work and had returned to work at the second interview with him, although he still suffered massive symptoms and had unresolved social and financial problems. At the third interview he was no longer working, as he felt unable to turn up for work, and had been to a psychiatric emergency room as he had considered committing suicide.

A few participants, on the other hand, felt that the interval before return to the labour market was too long. A person sick-listed with depression found participation in a psycho-educative group session both meaningful and useful. But as it was her first experience with sickness absence, she found herself in a vacuum without the job that used to give structure to her everyday life. At the third interview with her she complained that having to wait so long for the job training she had just started had made her insecure:

You feel that you are sort of beginning to slip. If I had started job training I don’t think there would have been any problems. Then I might just have started 15 h. But they were pretty slow to find out that I could do this [job training].” She describes the effect of her sickness absence on her perception of herself: “I have changed a great deal. I’m not the sort of hello-let’s-get-going person I used to be. But maybe I will be some day. (Interview 3 with participant 5, woman, 59, on sick leave)

When the job training was arranged she started 20 h a week and within a month she worked full time. For other participants it was stress-inducing to have to participate in the minimum 10-h-per-week mandatory RTW activities. They felt that neither their health nor their energy level allowed them to fulfill this requirement. All in all there seemed to be a wide variation in the participants’ need for intervention, timing of intervention and extent of intervention.

Below we will present our findings in relation to our third research question on the working mechanisms of the RTW program in order to illustrate the underlying processes and conditions that seem to impact the participants’ experience and the possible influence of participating in assessment consultations and RTW activities. We have chosen the term ‘working mechanism’ knowing that it is an analytical reduction of the complex and dynamic reality that faces the participants in their RTW-process. Our analysis indicates that three underlying conditions are essential (1) Individual approach (2), RTW professionals as legitimate experts, (3) Multidisciplinarity.

#### Working Mechanisms of the RTW Program

##### Individual Approach

Several participants described how ‘ being seen’ and ‘being met’—or the opposite—was decisive for whether the RTW program was experienced as useful and relevant or not. If the participants felt that the RTW professionals focused on them as unique persons with specific problems there was a clear tendency for possible resistance to and skepticism of assessment and RTW activities to be minimized. One participant with depression described the assessment consultation with the RTW coordinator:

…she [the RTW coordinator] saw me as a human being and not only as a statistic. She saw me, not as a case, but as an individual who needed some kind of help. Then I thought: “Oh my God. Here is somebody who thinks differently, somebody who thinks: Okay, you are not only my statistics…… I was completely taken aback, I was so happy. I was overjoyed and shed a tear when I left her. (Interview 2 with participant 9, man, 31, on sick leave).

This approach to the sick-listed person can be described as taking an ‘individual approach’. The participants characterized the RTW professionals that applied this approach as *attentive, interested, open*-*minded, reflective, empathic and sympathetic.* Feeling ‘met’ by an individual approach was perceived to be essential for participants to open up and describe their difficult and emotionally exhausting situation during assessment consultations. Without confidence and openness the RTW professionals would not get the necessary information during the assessment consultations, information which was considered important to form a ‘true’ picture of the sick-listed persons’ CMD and challenges and resources for RTW.

Not all participants had been in contact with RTW professionals who favoured an individual approach. One participant, sick-listed with depression, narrated his experiences with the RTW coordinator and the RTW team:

It doesn’t seem as if they spent very much time asking questions about how it, I mean like…they don’t ask: “What is it like being sick for a person like you?” or: “What is your health problem?” Because they don’t treat me as an individual. I would have liked that. That they sat down and said: “Now, tell us what you think is the matter with you? (Interview 2 with participant 8, man, 38, off the sick list but unemployed).

Regardless of whether the participants felt they had been met with an individual approach or not, it is a significant finding that every single one expressed a strong need for the RTW professionals to focus on them as concrete and unique individuals and to show genuine interest in them, their situation, their needs and their RTW-process.

##### RTW Professionals as Legitimate Experts

For the RTW professionals to be able to effectively intervene through RTW activities and influence the participants’ perception of their health problem, their symptoms and the compatibility of the job with these, they had to achieve a position as *legitimate experts* in the RTW-process of the sick-listed person. This position, however, was not always achieved at the first consultation. One person sick-listed with stress illustrated how she didn’t see the RTW professionals as legitimate experts at the assessment consultations:

… I mean…come on, man, I am sick-listed with stress - don’t invade my life! Or the thing about perfect strangers all of a sudden gaining access to some intimate parts of my life. I think: Go away! (Interview 3 with participant 4, woman, 47, partial RTW).

Another participant sick-listed with stress described her first meeting with the RTW professionals:

It can be totally intimidating to have to tell somebody you don’t know how terrible you feel. And be like totally honest about it. So they may think you are much better than you really are. So, in a way, it is like you are trapped. (Interview 2 with participant 16, woman, 26, off the sick list but unemployed)

The RTW professionals had to gain the confidence of the participants before being assigned the position as legitimate partners whereby the participants experienced that the professionals’ questions, interpretations and interventions were relevant and challenged their illness perception, motivation and initiatives aimed at RTW.

##### Multidisciplinary

Several participants mentioned that the multidisciplinary coordination made them feel confident that the RTW professionals together included as many aspects of their case as possible. A few participants even noticed that the RTW coordinator replaced a rather patronizing, impersonal approach to them with a more individual approach after discussing their case with the other RTW professionals at the weekly multidisciplinary conference. One participant sick-listed with stress experienced a noticeable difference between the first and second consultations with the RTW coordinator and commented on the difference:

- Participant: *“But, I mean, the way she approached me* [at the second consultation]*…I wasn’t at all on the defensive. And I totally dropped my guard. And then we actually had a constructive dialogue instead of her coming at me like: “now listen to me, this is unacceptable, now you just have to start working.” So it was a complete paradigm shift… … I had the feeling that at the first meeting she was a janitor watching the children playing in the backyard, ready to jump on them if they made a mess. If they did she would tell them to get out of the yard”*
- Interviewer: *“Okay. First she was a janitor, what was she at the second consultation?”*
- Participant: *“Well, she was much more, like…the kind janitor. Who took her time to sit down and play with the children in the sandbox. And not only…I mean, although she knew that she had to clean the mess …she still took her time.”* (Interview 2 with participant 1, man, 39, partial RTW)

At the second meeting the RTW coordinator had explicitly informed the participant that she had changed her mind about his situation and need for help after discussing his case with the other RTW professionals. The quote shows both how important an individual approach is and how multidisciplinarity can facilitate the application of it. Likewise the quote illustrates that the participant still perceives the RTW professional as an authority even though she practices the individual approach (the RTW coordinator is also described as a janitor *after* the paradigm shift). A central difference between his experience of the first and the second consultation is that the relation is no longer based on pressure and coercion. These have been replaced by a fruitful—if not equal—dialogue about a well-defined goal—RTW.

## Discussion

The aim of this qualitative study was to explore how persons on sick leave due to CMD experienced participating in the RTW program and how the workability assessments and RTW activities influenced their RTW-process. Conducting three interviews with each participant over an extended period of time gave us the possibility to look into and make hypotheses on relationships between different themes and subjects. We found that about half of the interviewed participants experienced that the workability assessments identified their need for intervention. Besides they had the potential to convey to the persons on sick leave the feeling that a qualified multidisciplinary unit guided their RTW-process competently. However, the consultations also had the potential to frustrate and confuse the participants. Overall, the participants experienced profiting from the offered RTW activities—if these were judged adequate—and especially psycho-education group sessions seemed to enhance the participants’ feeling of readiness for RTW. In relation to our third research question concerning the working mechanisms and underlying dynamics of the RTW program we have identified the individual approach as necessary for the realization of the potentials in the RTW program. Based on our results we hereby suggest a definition of the individual approach as the ability of the RTW professionals to both inspire in the sick listed worker the feeling of being met on his or her premises and of being seen as a unique individual and at the same time to exert their authority in the RTW-process—in accordance with the legal context of the RTW program described in the section Setting. Our results show that the nature of CMD and the experience of being sick-listed with CMD call for special attention to how the assessment consultations are introduced and conducted. The individual approach appears to be a precondition for the sick-listed persons to feel confident and motivated to communicate the necessary information by means of which the RTW professionals can make realistic workability assessment as the individual approach seems to reduce the demonstrated difficulties of participating in the consultations. In the result section we have described how participants experienced difficulties verbalizing and explaining their mental condition to the RTW professionals. This experience, however, could very well go beyond the very incapacity of verbalization. It might as well be related to stigmatization of psychiatric conditions [9] which may lead the participants to doubt the social legitimacy of their mental health problems. Our analyses indicate that the individual approach increases the trust in the RTW professionals and it can be seen as a precondition for the participants’ perception of the RTW professionals as legitimate partners and significant facilitators in the participants’ RTW-process. When the RTW professionals are established as legitimate partners they are in a position to influence positively the participants’ symptom level and belief in their own capability of returning to work. The combination of the individual approach and multidisciplinarity seems to be fundamental to a positive experience of the RTW intervention: The individual approach induces the feeling of being taken care of as a unique person with specific problems, and multidisciplinarity can inspire confidence that the complexity of one’s unique situation is embraced. Besides, multidisciplinarity seems to facilitate the application of the individual approach.

**Fig. 2:**
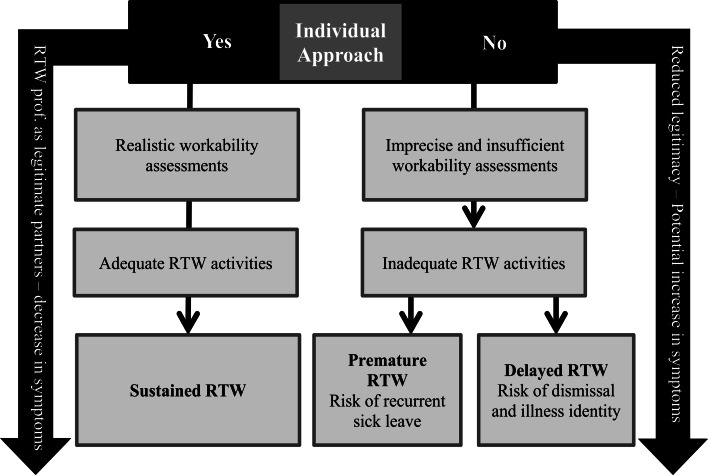
Potential relationship between the Individual approach and possible outcomes of sick leave

Based on our results and analyses a hypothesis can be formulated: If the persons on sick leave do not feel approached as individuals, the assessment consultations tend to not adequately identify barriers and resources for RTW. This may result in categorization of the participants as either more ready for work or less ready for work than is the actual case, which can lead to the sick-listed person being offered inadequate RTW activities. The impending risk is that the sick-listed person either returns to work prematurely and without relevant work modifications or endures prolonged status as sick-listed in the RTW program, thereby risking being fired or developing an undesirable illness identity. If, on the other hand the persons on sick leave receive adequate RTW activities they are more likely to return to work at the right time, at the right pace and with the proper workplace modifications. Figure 2 illustrates our hypothesis on how the presence or absence of the individual approach may affect the outcomes of participation in the RTW program. Our suggestion of an association between the individual approach and the three outcomes need to be further explored, tested and validated in future research. The knowledge gained herby could enhance and nuance our understanding of the mechanisms and the results of RTW interventions aimed at workers sick listed with CMD.

The results presented in this article are not meant to suggest that we can explain the outcome of a sickness absence by looking only at the quality of the relationship between the RTW professionals and the person on sick leave. The RTW-process is complicated and research shows that the medical seriousness of the disorder, work-related factors, personal factors, national compensation policies, and the structure of the health care system influence whether a sick leave results in return to or withdrawal from the labour market [7, 45–50]. While acknowledging this complexity, we want to further elaborate on the importance of the individual approach and the quality of the relation between the sick-listed person and the RTW professionals as these seem to be key factors in the participants’ experience of participating in the RTW intervention.

Other studies (mostly including workers with musculoskeletal disorders) have shown that how the persons on sick leave experience their encounters with rehabilitation professionals (Health Care, Social Insurance Agency) influences the outcome of a long term sick leave and the self-estimated ability and motivation to return to work [14, 51–56]. Being treated with respect, feeling supported, seen, heard and recognized as a person by the professionals were associated with promoting RTW [51, 52, 54], whereas negative experiences with rehabilitation professionals such as being treated in an indifferent, nonchalant or fairly routine manner resulted in a feeling of shame in persons on sick leave [56]. Respondents with psychiatric diagnoses more often reported on negative encounters with both healthcare providers [53, 57] and professionals within social insurance offices [58] compared to respondents with somatic disorders.

It has been argued that there is a need for theoretical contextualization and development of theoretical concepts to grasp the importance of the relationship between the sick-listed person and the health professionals [54, 59]. We will here briefly offer a contextualization by drawing upon theories and research from the field of psychotherapy to further explain and explore the findings of the qualitative study reported in this article.

Even though the RTW professionals are not allowed to offer traditional psychotherapy, the RTW program shares some features with therapy such as modification of the participants’ illness perception, developing more appropriate coping strategies and increasing self-efficacy [20, 22]. In recent years a number of reviews have been published on factors and conditions that can explain the effectiveness of psychotherapy [60, 61]. Decades of research have revealed that the *specific therapeutic method and techniques* can explain approximately 15 % of the variance of the outcome, the *patients’ expectations* of the therapy can explain 15 % of the success or failure, *extra*-*therapeutic change* (such as spontaneous remission, social support, fortuitous events) accounts for 40 % of the success. The last 30 % can be ascribed to *common factors* (therapeutic alliance, client factors and therapist factors) [62]. The concept “common factors” refers to aspects of psychotherapy that are present in most, if not all, approaches to therapy [60, 61, 63].

In relation to our illumination of the importance of the individual approach, and the quality of the relation between the person on sick leave and the RTW professionals, we find the research on therapeutic alliance and therapist factors to be of great relevance. Research on psychotherapy has shown that the therapeutic alliance is one of the strongest and most robust predictors of success or failure in psychotherapy [62, 64] and some have even argued that the therapeutic relationship is “the necessary and sufficient condition” in therapy [65]. There is also evidence that there is a positive correlation between therapist factors such as empathy, warmth, openness, positive regard, being nonjudgmental, attentiveness and the outcome of psychotherapy [66]. The theoretical explanation that the therapeutic alliance and therapist factors are essential to the outcome is that the focus of therapy is frequently on painful problems involving shame and guilt. If the alliance is of low quality, it will be difficult for the client to explore these problems with the therapist [67].

Research on common factors seems to validate and explain our results. Our study has shown that the relation between the sick-listed person and the RTW professionals and therapist factors are of great importance to participants with CMD’s experiences of the RTW intervention. Another qualitative study found that clinician-patient agreement about the work disability problem (persistent pain) and a high-quality therapeutic relationship was a precondition for exposure to work [68]. Even though the common factors might have great explanatory effect on the RTW outcome, there is no tradition for addressing these components and factors in quantitative evaluation of RTW interventions. There seems to be a potential in looking deeper into the quality and type of alliance and therapist factors when evaluating RTW interventions. Maybe this can give us a better understanding of why some RTW interventions manage and others fail to reduce the time to RTW. However, whether the results from research on psychotherapy are directly transferable to RTW interventions or whether there are different connections between the alliance and therapist factors and the outcome of RTW interventions should be further investigated. Even though some of the methods and techniques used in traditional therapy and RTW interventions are identical there is a substantial difference. In relation to the significance of the individual approach and the quality of the relation between the person on sick leave and RTW professionals, it is essential to be aware that the Danish legal context within which the RTW professionals operate may in itself complicate the use of the individual approach. For example, the fact that the RTW coordinator has to function as both facilitator and authority may complicate the creation of an open, confident and dignified relation between the sick-listed person and the RTW coordinator. Other researchers have pointed out that if the RTW agenda is a therapist-driven goal this may present an ethical dilemma and potential barriers to the development of a good therapeutic alliance, and it has been emphasized that there is a need for research on how the RTW agenda is best incorporated into the necessary collaborative relationship and therapeutic alliance [69]. Besides, we need to be aware of the fact that system-related conditions such as lack of resources, time and schedules can also be barriers to a good alliance as these can lead to unintended intimidation and humiliation of the client [70].

Our results indicate that the individual approach is important to decide on and tailor RTW activities matching the needs and work situation of the specific sick-listed person thereby increasing the chances of an early and sustainable RTW. This finding is in line with research on psychotherapy documenting that customizing the therapy to the patient increases the effectiveness of psychotherapy [71] and in line with research into occupational rehabilitation stressing that the rehabilitation intervention needs to be adjusted to the client’s needs to enhance the chances of RTW [72]. In the literature this practice has been referred to as “client-centred practice”. Client-centred practice is characterized by involving the client in decision making and ensuring that the intervention offered fits the client’s needs and context [72, 73].

Svensson, Müssener and Alexanderson [59] raise an important question to be answered: How can rehabilitation efforts be organized and structured to enhance positive social emotions and psychological empowerment of sickness absentees? Our study has indicated that the individual approach is a necessary—but not necessarily sufficient—ingredient in a rehabilitation and RTW program. As described in the section “Being on sick leave due to CMD” several participants were ashamed of their CMD and saw it as a personal defeat to have to report sick. This led them to a feeling of existential disturbance of identity. The individual approach could be a way to reduce the existential disturbance and start the process of enhancing positive emotions and psychological empowerment.

### Strengths and Limitations of this Study

A significant strength of this study is that the participants were recruited for the interviews before commencing the RTW project. We believe that we have thereby avoided a typical kind of selection bias e.g. that those who volunteer as interviewees have very strong opinions about and emotions in relation the subject under scrutiny. We furthermore find a significant strength in the fact that the participants were followed while they experience the process, which counters a tendency to recall bias, if participants are interviewed months or years after taking part in an intervention. One limitation of the study concerns the unexamined relationship between participant characteristics and RTW-process. The research into common factors, which we have drawn upon, has documented a relation between client characteristics (e.g. patient preferences, coping styles, stages of change, personality dimensions, and culture) and therapy outcome [71]. As we have only interviewed 17 participants, it has not been possible for us to go into details within this area. In relation to the subjects discussed in this article we find that we have reached acceptable data saturation as the last cases analyzed did not add significant new information relevant to answering the three research questions. The legal context is important in relation to RTW [14] and therefore one should be careful about uncritically transferring the results of this study to other countries as their legal context can differ from the one reported here. We do, however, believe that our study has revealed some fundamental aspects of being on sick leave with CMD and participating in similar RTW programs and that these aspects can be generalized and transferred to other western countries. This study has focused on sick listed workers with CMD and it is for future research to explore to which degree our results are transferable to other medical conditions. Our research design has given us the chance to explore in depth the experience of being on sick leave with CMD and participating in an RTW program. The conduction of three interviews with each participant had the advantage that potential vagueness in one interview could be explored and clarified in the following interview and the longitudinal research design also gave us the possibility to present some of the initial hypotheses to the participants. We do, therefore, believe that we have attained a reasonably good fit between the participants’ view of their situation and our representation of it.

### Implications for Practice and Research

We believe that we need to focus on the working mechanisms in RTW interventions to enhance the chances of positive RTW outcomes and we need research on the association of common factors to the outcome of RTW interventions. This research could be mixed method inspired by research methods such as quantitative process evaluation in psychotherapy [74] and principles from the qualitative method “Pragmatic case studies”, which has been developed specifically for understanding the working mechanisms of therapy and interventions [75–77]. This could give us the knowledge of differences and similarities between the individual approach and the therapeutic alliance. Likewise it could illuminate if and how specific sickness insurance regulations in themselves can reduce the professionals’ possibilities for practicing the individual approach and customizing the RTW interventions—and how this potential conflict can be managed. To look further into how the possible role conflict related to RTW professionals being both facilitators and authority could impact the professionals’ possibility of practicing the individual approach we would also like to suggest research from the perspective of the RTW professionals. When we have gained more knowledge on the potential dilemmas and role conflict experienced by the professionals and on which common factors can increase the quality and effect of RTW interventions for persons sick listed witch CMD, this knowledge ought to be used to educate and train rehabilitation professionals in skills and capabilities associated with practicing the individual approach and also create conditions that reduce the potential role conflict of the RTW professionals.

## Conclusion

To date, there has been little research on how persons on sick leave due to CMD experience participating in an RTW intervention and how specific elements in the intervention influence their RTW-process. This study contributes to existing knowledge on RTW by exploring three elements which are common in RTW interventions: Workability assessments, psychological interventions (in group or individual sessions) and the general working mechanisms of the intervention. We have shown that the assessment consultations have the potential to result in both motivation and frustration, and three overall challenges in relation to the assessment have been identified. Our results indicate that psycho-educational group sessions have the potential to transform illness representations and increase readiness to RTW whereas individual sessions with a psychologist are mostly helpful for sick-listed persons with less severe social, health- and work-related problems. We have illuminated how the individual approach seems necessary for the realization of the positive potential in the RTW program. However, the fact that the RTW professionals are both the helpers and the authorities in the sick-listed persons’ RTW-process is an inherent paradox in the RTW program, which can impede the establishment of a high-quality relationship between the sick-listed persons and the RTW professionals. We have suggested that researchers and practitioners in the field of RTW interventions take inspiration from research on therapeutic alliance and therapist factors when designing and evaluating RTW interventions. More research is needed on which types of alliance, therapist factors, and client factors are associated with a successful outcome of an RTW intervention and RTW practitioners should be trained in relevant interpersonel competencies and be provided with optimal conditions to put these into practice.
